# Editorial: A new perspective in immune polymorphism (the HLA, KIR, and LILR genes)

**DOI:** 10.3389/fimmu.2025.1640672

**Published:** 2025-06-18

**Authors:** Seik-Soon Khor, Martin Maiers, Jason Krawic, Danillo G. Augusto, Aisha Souquette

**Affiliations:** ^1^ Singapore Centre for Environmental Life Sciences Engineering, Nanyang Technological University, Singapore, Singapore; ^2^^®^ CIBMTR (Center for International Blood and Marrow Transplant Research), NMDP, Minneapolis, MN, United States; ^3^ Department of Microbiology and Immunology, The University of Oklahoma Health Sciences Center, Oklahoma City, OK, United States; ^4^ Department of Biological Sciences, The University of North Carolina at Charlotte, Charlotte, NC, United States; ^5^ Department of Microbiology & Immunology, University of Maryland School of Medicine, Baltimore, MD, United States

**Keywords:** HLA, KIR, LILR, LIBAG, HIV, PyPop, MICA, the society for immune polymorphism

The polymorphism within the *HLA*, *KIR (killer-cell immunoglobulin-like receptor)*, and *LILR (leukocyte immunoglobulin-like receptor)* gene families profoundly influences the evolution of the human immune system. This genetic polymorphism influences disease susceptibility, immune regulation, evolutionary mechanisms, and drug response. This Research Topic brings together a collection of the latest research that delves into the complexity of these gene families, providing novel insights into their associations with diseases and the fundamental knowledge for future investigation.

This Research Topic features three papers exploring HLA associations with distinct diseases—HIV(Rahmouni et al.), dengue (Ghosh et al.), and type 1 diabetes (Noble et al.). Using data from the International Collaboration for the Genomics of HIV, Rahmouni et al. revisited the association of SNPs (single nucleotide polymorphisms) and *HLA* alleles with HIV-1 elite control. This study reported that HLA-B*57:01, which is part of a very large HLA haplotype block, is significantly associated with HIV-1 elite control in European and African American cohorts. The authors suggested that, in addition to the conventional antigen-presenting role of HLA molecules, HLA class I may provide an alternative molecular mechanism for HIV-1 elite control that involves changes in immune gene expression that could be mediated by transcription factors encoded in this haploblock. Meanwhile, considering the dynamic climate change-associated expansion of vector-borne diseases, Ghosh et al. examined the association of *HLA* alleles with different stages of dengue across worldwide populations. This review (2) highlighted the complex, often contrasting, and varied nature of HLA-dengue associations worldwide, eliciting the urgent need for further studies among understudied populations to inform public health strategies and combat this emergent threat. Additionally, research by Aharon et al. into macrophage migration inhibitory factor (MIF) functional polymorphism revealed associations with the progression of acute graft-versus-host disease (GVHD) and steroid refractoriness in patients who underwent allogeneic hematopoietic stem cell transplantation (HSCT). The review of HLA-associated type 1 diabetes risk by Noble et al. summarizes work that has evolved since the mid-1970s, presents modern association analysis strategies, and suggests that, while traditional antigen presentation by HLA remains the strongest contributor to disease risk, additional mechanisms are likely to modify risk.

The LILR genes encode for both inhibitory and activating receptors on myeloid and lymphoid cells and are a highly understudied component of the immune system. Hirayasu et al. reported a novel hybrid gene between *LILRB5* and *LILRB3*, namely *LILRB5-3*. The hybrid gene was detected using the JoGo-LILR (1) tool from a combination of short-read whole genome sequencing (srWGS) and a dataset generated with long-read sequencing. Another study from the same group (Li et al.) demonstrated that fibrinogen induces inflammatory responses via the immune-activating receptor *LILRA2* gene, and may represent a novel therapeutic target for inflammatory disease. On a similar note, Khor et al. introduced the development of the software *LILR* genotype imputation with attribute bagging (LIBAG), which enables copy number imputation for *LILRB3*, *LILRA6*, and *LILRA3*. This approach will enhance the ability to study LILR gene variations and their associations with diseases.

Similarly, a paper by Lancaster et al. describes the major release of Python for Population Genomics (PyPop) version 1.0.0. This specialized software package is designed to process *HLA* genotype and allele data for large-scale population genetic analyses, specifically for highly-polymorphic gene systems. A highlight of PyPop 1.0.0 is the addition of the asymmetric linkage disequilibrium (ALD) test to the package’s analytic suite. ALD extends historical LD measures, which assumed equal numbers of variants at each locus, for highly polymorphic gene systems, which frequently display different numbers of variants at each locus.

Klussmeier et al. investigate the prevalence and origins of *MICA* (MHC class I polypeptide-related sequence A) gene copy number variations (CNVs) in more than 2 million individuals. The study found that high-frequency CNVs result from independent nonallelic homologous recombination events between segmental duplications upstream of *MICA* and *MICB*.

Lastly, Mora-Bitria and Asquith provide a comprehensive review of how inherited natural killer (NK) cell receptors, particularly KIR molecules, influence T-cell responses. The authors discuss the mechanisms, including NK cell-mediated elimination of activated T cells and the modulation of antigen-presenting cells.

## 1 The society for immune polymorphism

The Society for Immune Polymorphism (SIP) is a global scientific organization dedicated to advancing research on the diversity of the vertebrate immune system and its implications for evolutionary biology, health and disease. The SIP’s activities encompass a range of initiatives aimed at fostering collaboration and disseminating knowledge in the field of immune polymorphism.

### 1.1 Scientific symposia

The SIP organizes an annual international symposium that serves as a platform for researchers to present and discuss their research and establish new collaborations. The next symposium is scheduled for Nov 3-5, 2025 in Sassenheim, the Netherlands. This event will feature lectures and discussions on polymorphic immune molecules and their functional diversity across species, contributing to the advancement of knowledge in this field (http://sip-sassenheim.org).

### 1.2 Educational webinars

The SIP hosts webinars that focus on specific aspects of immune polymorphism, providing educational content to the scientific community. These webinars are accessible through its website and cover a range of topics pertinent to researchers and clinicians interested in immune system diversity.

### 1.3 Research collaborations and resources

The SIP facilitates international collaborations among researchers studying immune polymorphism, providing resources and support for studies that explore the genetic variations in immune-related genes and their associations with diseases. This includes the development of data standards, tools, and databases that aid in the analysis of immune system diversity. This Research Topic is the second collection organized by SIP, following a set of publications on HLA and KIR diversity and polymorphism, published in 2021 (2).

### 1.4 Advocacy and public engagement

The SIP engages in advocacy efforts to raise awareness about the importance of investigating immune polymorphism. The society aims to influence public health policies and funding priorities to support research in this critical area. Additionally, the SIP works to engage the public and policymakers in understanding the significance of immune system diversity in health and disease.

For more detailed information on the SIP’s activities and upcoming events, visit http://www.immunepolymorphismsociety.org.
